# Pregnancy Outcomes in Thyroid Cancer Survivors: A Propensity Score-Matched Cohort Study

**DOI:** 10.3389/fendo.2022.816132

**Published:** 2022-02-17

**Authors:** Qi Cao, Huili Zhu, Jiani Zhang, Yujing Li, Wei Huang

**Affiliations:** ^1^ Department of Obstetrics and Gynaecology, West China Second University Hospital of Sichuan University, Chengdu, China; ^2^ Key Laboratory of Birth Defects and Related Diseases of Women and Children (Sichuan University), Ministry of Education, West China Second University Hospital of Sichuan University, Chengdu, China; ^3^ Key Laboratory of Chronobiology (Sichuan University), National Health Commission (NHC), West China Second University Hospital of Sichuan University, Chengdu, China

**Keywords:** thyroid cancer, thyroidectomy, radioiodine therapy, gestational thyroid function, pregnancy outcomes

## Abstract

**Background:**

Some female thyroid cancer survivors wish to become pregnant following their cancer treatment. Current studies have shown inconsistent results on pregnancy outcomes in these survivors; however, detailed information on the pathological type, treatment, and gestational thyroid function of these patients are not yet well documented, making the refined assessment of the influence of a history of thyroid cancer and related treatments on pregnancy outcomes challenging.

**Objective:**

To investigate the risk of adverse pregnancy outcomes in thyroid cancer survivors.

**Methods:**

This was a retrospective cohort study. We included all women aged between 19 and 45 years old who delivered between January 2019 and June 2020 in West China Second University Hospital of Sichuan University. Women with tumors other than thyroid cancer or other thyroid diseases were excluded. The included women were divided into survivors of thyroid cancer (survivors) and women without any history of thyroid disease (controls). Propensity score matching and logistic regression were used to control confounding variables.

**Results:**

All 18,332 women who met the inclusion criteria were included in the study (96 survivors of papillary thyroid cancer and 18,236 controls). After propensity score matching, 96 survivors and 192 controls were included. The survivors had higher levels of free thyroxine (15.47 [13.61–17.67] vs. 14.38 [13.20–15.81] pmol/mL; P<0.001) and higher levels of thyroid peroxidase antibodies (TPOAb) (43.55 [31.43–71.43] vs. 35.95 [28.00–48.03] U/mL; P=0.008) but similar levels of thyroid stimulating hormone (1.46 [0.56–3.15] vs. 1.36 [0.81–1.92] mIU/mL; P=0.142) than the controls. There were no significant differences in adverse pregnancy outcomes between survivors and controls. Fetal macrosomia was lower among survivors (OR: 0.077, 95% CI: 0.009–0.668. P=0.020) than controls. Additionally, survivors had reduced weight gain during pregnancy (13.0 [10.0–15.0] vs. 14.00 [11.00–16.00] kg, P=0.005) and reduced placental weight (563.0 [514.5–620.0] vs. 572.0 [520.0–650.0] g, P=0.019), albeit with small absolute differences. Thyroidectomy or radioiodine therapy did not adversely affect pregnancy outcomes.

**Conclusion:**

A history of treated papillary thyroid cancer was not associated with adverse pregnancy outcomes.

## Introduction

Thyroid cancer has become the most common malignant endocrine cancer among women of childbearing age, and patients usually have a favorable prognosis (1–5). Some female survivors wish to become pregnant following their thyroid cancer treatment. However, existing thyroid cancer-related treatments may have adverse effects on pregnancy outcomes.

According to the current guidelines, thyroidectomy with or without radioiodine therapy (RAIT) and postoperative thyroid-stimulating hormone (TSH) suppressive therapy with levothyroxine (LT4) are the standard treatments for thyroid cancer (5–7). During pregnancy, thyroxine is vital in maintaining maternal health and fetal development (7, 8). However, both hypothyroidism caused by thyroidectomy and hyperthyroidism after TSH suppressive therapy may lead to adverse pregnancy outcomes, including pre-eclampsia, gestational diabetes mellitus, preterm delivery, and miscarriage (9–11). In addition, studies have shown that RAIT might lead to gonadal dysfunction and follicular atresia (12). Impaired gonadal function may contribute to adverse pregnancy outcomes, especially miscarriage.

Two national database studies have shown different results. A Korean research study by Cho et al. found a higher risk of postpartum hemorrhage (PPH) among survivors (13). A US-based study by Spiegel et al. showed a higher incidence of thromboembolism and blood transfusion among survivors (14). The other three smaller-sized studies (15–17) generally showed no adverse fetal outcomes. However, PPH was mentioned in Clark et al.’s study (17). Detailed information on the pathological type, treatment, and gestational thyroid function of thyroid cancer survivors are still not well understood, making the refined assessment of the impact of a history of thyroid cancer and related treatments on pregnancy outcomes challenging.

Therefore, we conducted this retrospective cohort study among women with treated thyroid cancer to evaluate the gestational thyroid function of the survivors and determine the potential adverse maternal or fetal outcomes of thyroid cancer-related treatments.

## Materials and Methods

### Study Design

This study was approved by the Ethics Committee of West China Second University Hospital of Sichuan University (No. 2021098) and performed in accordance with the tenets of the Declaration of Helsinki. Informed consent was not required as we analyzed data anonymously without divulging any personal information.

Excluding those with a history of other tumors or other thyroid diseases, we included all women aged between 19 and 45 years who delivered between January 2019 and June 2020 in West China Second University Hospital of Sichuan University. The group was divided into survivors and controls based on the presence or absence of a history of thyroid cancer, respectively.

Baseline information, pregnancy outcomes, and neonatal data were obtained from the electronic medical records (EMR) of West China Second University Hospital of Sichuan University. Thyroid cancer-related data including pathological type, treatment modality, and duration were collected with patients’ consent from the EMR of the hospital where the patients were diagnosed and/or treated. Patients’ blood samples were routinely collected between 0800 and 1130 hours. Thyroid function tests including TSH, free thyroxine (fT4), and thyroid peroxidase antibodies (TPO-Ab) were performed between gestational weeks 10 and 14. TSH, fT4, and TPO-Ab were measured in the laboratory of West China Second University Hospital of Sichuan University by chemiluminescence (Siemens ADVIA Centaur CP, Siemens Medical Solutions Diagnostics, Tarrytown, NY, USA). The reference range was 0.1–2.5 mIU/L for TSH (intra-assay coefficient of variation [CV]=2.1%, inter-assay CV=1.9%), 11.5–22.7 pmol/L for fT4 (intra-assay CV=2.22%, inter-assay CV=3.48%), and <60 U/mL for TPO-Ab (intra-assay CV=7.8%, inter-assay CV=7.3%).

### Outcome Measures

Maternal outcomes in this study included PPH (≥1000 mL for cesarean delivery or ≥500 mL for vaginal delivery); preterm delivery (between gestational weeks 28 and 36 + ^6^); pre-eclampsia (hypertension after week 20 with concomitant proteinuria); gestational diabetes mellitus (GDM, diagnosed according to a the 75-g oral glucose tolerance test between week 24 and 28, with plasma glucose thresholds for fasting, 1, and 2 h being 5.1, 10.0, and 8.5 mmol/L, respectively); late miscarriage (between gestational weeks 14 and week 27 + ^6^); cesarean section; anemia (hemoglobin<100 g/L); weight gain during pregnancy; and the blood pressure in the third trimester of pregnancy.

The neonatal outcomes of this study included macrosomia (newborn birthweight >4000 g); small for gestational age (SGA), i.e., birth weight below the 10th percentile for sex-specific gestational age; fetal death (after week 20); malformation; placental weight; birth weight; birth length; offspring sex; and Apgar score.

### Statistical Analysis

Continuous data were presented as the mean (standard deviation) for normally distributed data and as median (first quartile, third quartile) for non-normally distributed data. Categorical data were presented as percentages. Descriptive statistics were analyzed between groups by the independent samples *t*-test and the Mann–Whitney U test (if data were not normally distributed). Pearson’s chi-square test was applied for categorical data with the Fisher’s exact test for expected frequencies <5.

We used propensity score matching (PSM) to eliminate possible confounding factors and selection bias with SPSS 22.0 (IBM Corp., Armonk, NY, USA) embedded with the PSM plug-in (IBM Corporation, Armonk, NY, USA). A 1:2 matching was performed on the propensity score (PS) with a maximum caliber of 0.05. The PS was calculated using multivariable logistic regression models. Matching variables are presented in **Table 1**. We adjusted the *P-*values and odds ratios for the matching variables and other possible confounders by unconditional logistic regression models. Pre-pregnancy hypertension and diabetes mellitus were not involved as neither was present in both groups after PSM.

**Table 1:** Characteristics of the factors in propensity score matching.

	Survivor	Control			
		Before PSM		After PSM	
	(n = 96)	Control (n = 18236)	*P*	Control (n = 192)	*P*
Age (years)	32 (30-34)	31 (29-34)	0.032*	32 (29-34)	0.729
Ethnicities					
Han Chinese	96 (100%)	17625 (96%)	0.080	192 (100%)	>0.999
Other ethnicities	0 (0%)	611 (3%)	0.080	0 (0%)	>0.999
IVF	8 (8%)	1816 (9%)	0.596	12 (6%)	0.512
Gravida					
1	29 (30%)	7197 (39%)	0.064	59 (30%)	0.928
2	29 (30%)	5213 (28%)	0.726	66 (34%)	0.478
≧3	38 (39%)	5826 (31%)	0.110	67 (34%)	0.436
Parity					
0	59 (61%)	11609 (63%)	0.655	116 (60%)	0.864
1	32 (33%)	6178 (33%)	0.910	72 (37%)	0.488
≧2	5 (5%)	449 (2%)	0.084	4 (2%)	0.166
Pre-pregnancy condition					
Hypertension	0 (0%)	164 (0.85%)	>0.999	0 (0%)	>0.999
Diabetes mellitus	0 (0%)	190 (0.98%)	0.629	0 (0%)	>0.999

Data are presented as number (the percentage).

PSM, propensity score matching; IVF, in vitro fertilization.

*represents a statistical difference.

The selection of confounders is presented in directed acyclic graphs (**Supplementary Figures S1A–C**). Intermediate factors (fT_4_, TSH, TPO-Ab) were not adjusted to estimate the total effect of thyroid cancer (Model 1). FT_4_ and TPO-Ab were then included based on Model 1 to explore their effects (**Table 4**). All data were analyzed with SPSS 22.0. A two-tailed *P-*value <0.050 was considered to indicate statistically significant differences.

Subgroup analysis of treatment modality was performed between survivors and the corresponding PS-matched controls using regression analysis. Chi-square analysis and ANOVA analysis were performed to compare the pregnancy outcomes among the survivors with different treatment modalities.

In order to explore the impact of the time interval between conception and thyroidectomy, the survivors were trisected as Group T1, Group T2, Group T3. Logistic regression was used to calculate the P-trend across groups.

The study of time interval between conception and RAIT was not allowed due to the small sample size (n=33).

## Results

### Participants

**Figure 1:**
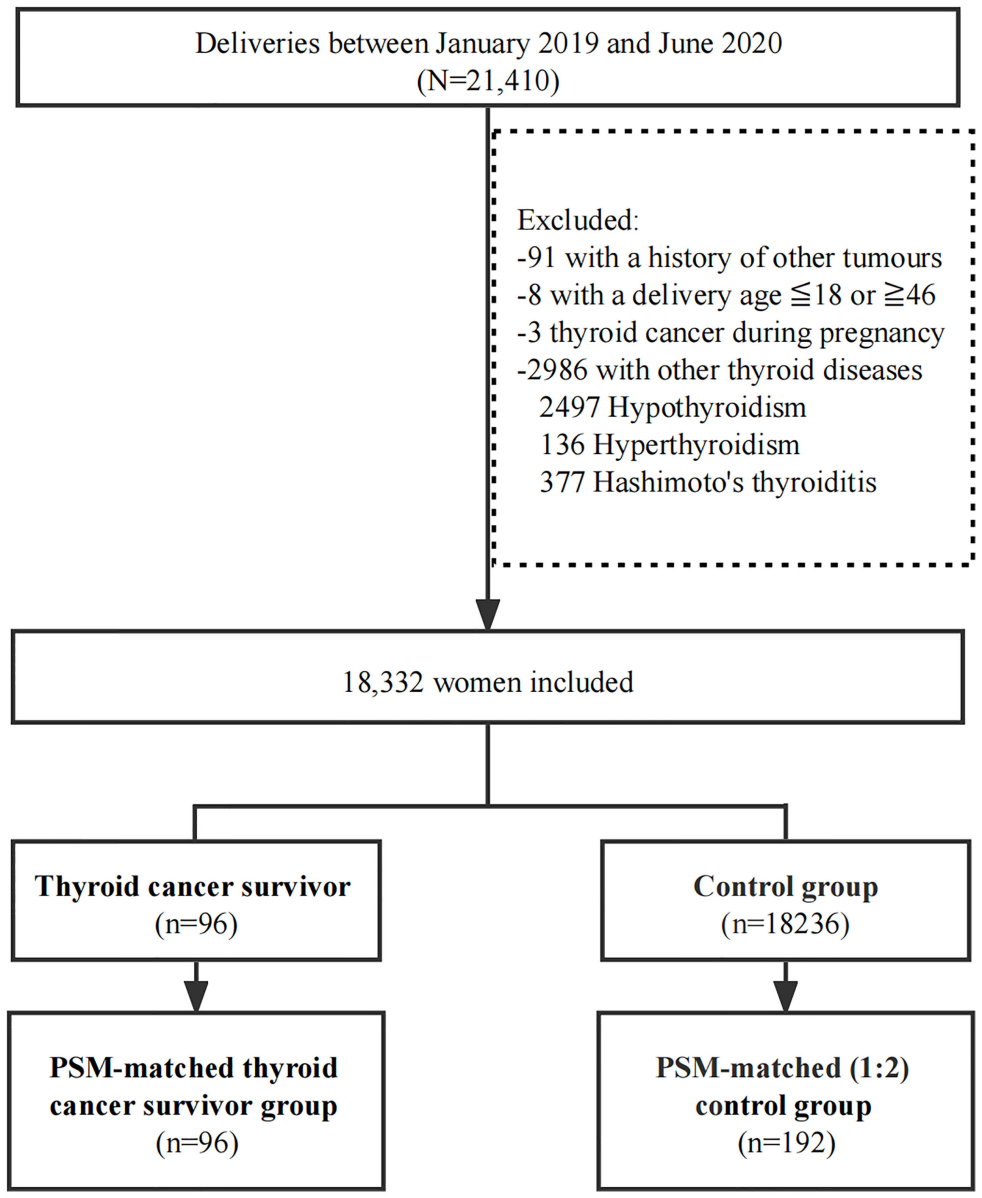
The participants enrollment. PSM, propensity score matching.

Between January 2019 and June 2020, 21,410 women gave birth in West China Second University Hospital of Sichuan University. Ninety-nine women had a history of thyroid cancer, but three of them were excluded for being diagnosed while pregnant. All 18,332 women who met the inclusion criteria were included in the study (96 in the thyroid cancer survivor group and 18,236 in the controls according to the subgroup criteria) (**Figure 1**).

A 1:2 PSM was performed to obtain matched controls (n=192). The baseline characteristics between groups were comparable after PSM (**Tables 1**, **2**).

**Table 2:** The baseline and thyroid function information of included women.

	Survivor (n = 96)	Control (1:2 PSM, n = 192)	*P*
Age (years)	32 (30-34)	32 (29-34)	0.729
Ethnicities			
Han Chinese	96 (100%)	192 (100%)	>0.999
Other ethnicities	0	0	>0.999
Gravida			
1	29 (30%)	59 (31%)	0.928
2	29 (30%)	66 (34%)	0.478
≧3	38 (40%)	67 (35%)	0.436
Parity			
0	59 (61%)	116 (60%)	0.864
1	32 (33%)	72 (38%)	0.488
≧2	5 (5%)	4 (2%)	0.166
IVF	8 (8%)	12 (6%)	0.512
Menstrual history			
Age of menarche	13 (12-14)	13 (12-13)	0.942
Menstrual duration	6 (5-6.9)	5.5 (4.5-6.5)	0.095
Interval between periods	72 (67-79)	73 (67-79)	0.512
Pre-pregnancy condition			
Hypertension	0 (0%)	0 (0%)	>0.999
Diabetes mellitus	0 (0%)	0 (0%)	>0.999
Weight (kg)	53.00 (50.00-56.50)	54.00 (50.00-60.00)	0.440
Height (cm)	161 (158-165)	160 (157-164)	0.774
BMI (kg/m^2^)	20.70 (19.03-22.61)	20.56 (19.50-22.58)	0.624
Gestational length at delivery (days)	274.5 (269-278)	273 (266-278)	0.313
Thyroid function in early pregnancy			
fT_4_ (pmol/ml)	15.47 (13.61-17.67)	14.38 (13.20-15.81)	<0.001*
TSH (mIU/ml)	1.46 (0.56-3.15)	1.36 (0.81-1.92)	0.142
TPO-Ab (U/ml)	43.55 (31.43-71.43)	35.95 (28.00-48.03)	0.008*
TPO-Ab≧60 U/ml	28 (29%)	24 (13%)	0.001*

Data are presented as median (first quartile, third quartile) or number (the percentage).

PSM, propensity score matching; IVF, in vitro fertilization; DM, Diabetes mellitus; BMI, body mass index; fT4, free thyroxine; TSH, thyroid-stimulating hormone; TPO-Ab, thyroid peroxidase antibodies.

*represents a statistical difference.

All 96 survivors were pathologically diagnosed with papillary thyroid cancer. Of these 96 women, 25% (24/96) received hemithyroidectomy, 41% (39/96) received total-thyroidectomy, and 33% (33/96) received total-thyroidectomy combined RAIT (**Table S1**).

There were statistical intergroup differences in fT4 and TPO-Ab. The survivors had higher levels of fT4 (15.47 [13.61–17.67] vs. 14.38 [13.20–15.81] pmol/mL; *P<*0.001) and TPO-Ab (43.55 [31.43–71.43] vs. 35.95 (28.00–48.03) U/mL, *P*=0.008] (**Table 2**). The incidence of abnormal TPO-Ab (≧60 U/mL) was also higher in the survivors (P<0.001) at 29% (28/96) vs. 13% (24/192). However, in both groups, the mean values of TPO-Ab were still within the reference range. No significant difference was found in TSH (1.46 [0.56–3.15] vs. 1.36 [0.81–1.92] mIU/mL, *P=*0.142].

### Maternal Outcomes

After PSM and the adjustment of potential confounders, no differences were found in the incidence of PPH (OR=0.429, 95%CI=0.090–2.039, *P*=0.287); preterm delivery (OR=0.439, 95%CI=0.155–1.243, *P*=0.121); pre-eclampsia (OR=2.779, 95%CI=0.708–10.91, *P*=0.143); and GDM (OR=1.126, 95%CI=0.609–2.084, *P*=0.705) (**Table 3**). The weight gain during pregnancy of thyroid cancer survivors was 1.0 kg lesser than that of the controls (13.00 [10.00–15.00] vs. 14.00 [11.00–16.00] g, *P*=0.005).

**Table 3:** Summary of the outcomes comparing thyroid cancer survivors and control.

					Model 1^#^	
	Survivor (n = 96)	Control (1:2 PSM, n = 192)	*Crude OR or MD *(95% CI)	*Crude P*	*OR _adjusted_* (95% CI)	*P_adjusted_*
**Maternal outcomes**						
Postpartum hemorrhage (≧1000 mL)^a^	2 (2%)	9 (5%)	0.433 (0.092-2.043)	0.347	0.429 (0.090-2.039)	0.287
Preterm delivery^a^	5 (5%)	22 (11%)	0.425 (0.156-1.159)	0.086	0.439 (0.155-1.243)	0.121
Pre-eclampsia^a^	5 (5%)	4 (2%)	2.582 (0.677-9.847)	0.281	2.779 (0.708-10.91)	0.143
Gestational diabetes mellitus^a^	21 (22%)	39 (20%)	1.098 (0.604-1.998)	0.758	1.126 (0.609-2.084)	0.705
Late miscarriage^a^	1 (1%)	1 (1%)	2.011 (0.124-32.496)	>0.999		0.979
Caesarean section^a^	66 (69%)	115 (60%)	1.473 (0.876-2.476)	0.143	1.404 (0.805-2.449)	0.232
Anemia^b^	10 (10%)	27 (14%)	0.711 (0.329-1.536)	0.383	0.668 (0.292-1.529)	0.340
Weight gain (kg)^b^	13.00 (10.00-15.00)	14.00 (11.00-16.00)	-1.00 (-2.00-0.00)	0.049*		0.005*
Blood pressure in third trimester of pregnancy^b^						
Systolic blood pressure (mmHg)	118 (109-125)	119 (111-126)	2 (-1-4)	0.228		0.207
Diastolic blood pressure (mmHg)	72 (67.3-79)	73 (67-78.8)	0 (-2-2)	0.963		0.957
**Neonatal outcomes**						
Macrosomia^b^	1 (1%)	16 (8%)	0.116 (0.015-0.887)	0.013*	0.077 (0.009-0.668)	0.020*
Small for gestational age^b^	2 (2%)	2 (1%)	2.021 (0.28-14.574)	0.603	2.676 (0.285-25.131)	0.389
Fetal death^a^	2 (2%)	2 (1%)	2.021 (0.28-14.574)	0.603	2.193 (0.298-16.119)	0.391
Malformation^c^	1 (1%)	1 (1%)	2.011 (0.124-32.496)	>0.999	3.137 (0.154-63.902)	0.457
Placental weight (g)^b^	563.0 (514.5-620.0)	572.0 (520.0-650.0)	10.0 (-12.0-32.0)	0.401		0.019*
Birthweight (g)^b^	3230.0 (2970.0-3515.0)	3240.0 (2900.0-3570.0)	20.0 (-100.0-150.0)	0.687		0.063
Birth length (cm)^b^	50.0 (48.0-51.0)	50.0 (48.0-51.0)	0.0 (-1.0-0.0)	0.794		0.843
Offspring sex						
Female	43 (45%)	104 (54%)	0.687 (0.42-1.123)	0.134		
Male	53 (55%)	88 (46%)	1.457 (0.89-2.383)	0.134		
Apgar score^b^						
1 min≦7	1 (1%)	2 (1%)	1.000 (0.090-11.168)	>0.999		0.998
5 min≦7	0 (0%)	0 (0%)				
10 min≦7	0 (0%)	0 (0%)				

Data were presented as median (first quartile, third quartile) or number (the percentage).

PSM, propensity score matching; OR, odds ratio; MD, mean difference; CI, confidence interval.

^#^Model 1, odds ratio and P value were adjusted for the matching variables and other possible confounders (The selection of confounders is illustrated in directed acyclic graphs in **Supplementary Figure S1**. Pre-pregnancy hypertension and diabetes mellitus were not involved because they were not present in either group after propensity score matching.): ^a^ adjusted for age, in vitro fertilization, gravida, parity, pre-pregnancy body mass index; ^b^ adjusted for age, in vitro fertilisation, gravida, parity, pre-pregnancy weight, height, body mass index, gestational length; ^c^ adjusted for age, in vitro fertilization, pre-pregnancy body mass index.

*represents a statistical difference.

### Neonatal Outcomes

For neonatal outcomes, placental weight was 9 g lighter in survivors than in controls (563.0 [514.5–620.0] vs. 572.0 [520.0–650.0] g, *P*=0.019] (**Table 3**). We also found a lower risk of macrosomia among survivors (OR=0.077, 95%CI=0.009–0.668, *P*=0.020) (**Table 3**). After further adjustment of fT_4_ and TPO-Ab (Model 2, Model 3, and Model 4), the differences were still significant (**Table 4**).

**Table 4:** Further adjusted *P-*value for thyroid function.

	*P _adjusted_*			
	Model 1^a^	Model 2^b^	Model 3^c^	Model 4^d^
Weight gain (kg)	0.005*	0.009*	0.041*	0.046*
Placental weight (g)	0.019*	0.025*	0.013*	0.033*
Macrosomia	0.020*	0.046*	0.032*	0.047*

a Model 1: P-value was adjusted for the matching variables and other possible confounders (The selection of confounder is illustrated in directed acyclic graphs in **Supplementary Figure S1**.): age, in vitro fertilization, gravida, parity, pre-pregnancy weight, height, body mass index, gestational length.

b Model 2: FT_4_ was included in the covariates on the basis of Model 1.

c Model 3: TPO-Ab was included in the covariates on the basis of Model 1.

d Model 4: FT_4_ and TPO-Ab were included in the covariates on the basis of Model 1.

*represents a statistical difference.

### Subgroup Analysis and Comparison of Different Treatment Modalities

No statistical differences in pregnancy outcomes were found in subgroup analysis between survivors receiving specific treatment modality and the corresponding PS-matched controls (**Table 5**). The comparison of the three treatment modalities found no differences in pregnancy outcomes as well (**Table 5**).

**Table 5:** Subgroup analysis and comparison of different treatments modality among thyroid cancer survivors.

	Subgroup analysis *β (95%CI) P-value/OR (95%CI) P-value*			*P* ^c^
	Hemi‐thyroidectomy without RAIT	Total-thyroidectomy without RAIT	Total-thyroidectomy combined RAIT	
	(24: 48 PSM-controls)	(39: 78 PSM-controls)	(33: 66 PSM-controls)	
**Maternal outcomes**				
Postpartum hemorrhage (≧1000 mL)^b^	–	–	2.06 (0.28, 15.35) 0.479	0.113
Preterm delivery^b^	–	0.51 (0.13, 1.94) 0.321	0.79 (0.14, 4.29) 0.782	0.219
Pre-eclampsia^b^	–	2.05 (0.28, 15.16) 0.480	6.50 (0.65, 65.10) 0.111	0.183
Gestational diabetes mellitus^b^	1.54 (0.43, 5.49) 0.504	1.15 (0.47, 2.80) 0.759	0.83 (0.28, 2.39) 0.724	0.740
A1^b^	2.89 (0.70, 11.98) 0.143	1.65 (0.63, 4.34) 0.310	0.69 (0.20, 2.36) 0.554	0.454
A2^b^	–	0.32 (0.04, 2.72) 0.294	1.35 (0.22, 8.53) 0.746	0.315
Late miscarriage^b^	2.04 (0.12, 34.16) 0.619	–	–	0.246
Caesarean section^b^	2.14 (0.72, 6.36) 0.170	1.24 (0.56, 2.75) 0.593	1.40 (0.57, 3.43) 0.458	0.656
Anemia^b^	1.00 (0.23, 4.40) >0.999	0.27 (0.06, 1.26) 0.096	1.29 (0.39, 4.32) 0.675	0.329
Weight gain (kg)^a^	-1.33 (-2.98, 0.32) 0.119	-0.61 (-2.14, 0.91) 0.431	-1.87 (-3.83, 0.10) 0.066	0.718
Blood pressure in third trimester of pregnancy (mmHg)				
Systolic blood pressure^a^	-5 (-9.90, 0.44) 0.078	0.4 (-3.91, 4.78) 0.844	-1 (-5.89, 3.01) 0.527	0.241
Diastolic blood pressure^a^	-2 (-5.87, 1.49) 0.248	2 (-1.74, 5.33) 0.322	0.1 (-3.66, 3.87) 0.956	0.192
**Neonatal outcomes**				
Macrosomia^b^	–	–	0.26 (0.03, 2.24) 0.222	0.340
Small for gestational age^b^	–	2.05 (0.28, 15.16) 0.480	–	0.160
Fetal death^b^	2.04 (0.12, 34.16) 0.619	–	–	0.338
Malformation^b^	–	–	–	0.246
Placental weight (g)^a^	-11.8 (-73.39, 49.72) 0.708	-18.0 (-65.98, 29.92) 0.463	-22.7 (-67.69, 22.33) 0.326	0.417
Birthweight (g)^a^	185.7 (-214.01, 585.47) 0.366	56.5 (-166.01, 279.02) 0.620	-218.9 (-435.83, -2.05) 0.051	0.483
Birth length (cm)^a^	1.9 (-1.46, 5.17) 0.276	1.6 (-0.60, 3.71) 0.161	-1.1 (-2.25, 0.01) 0.055	0.606

Subgroup analysis of treatment modality was performed between survivors and the corresponding PS-matched controls using regression analysis. The statistical results of continuous variables ^a^ were expressed as “β (95%CI) p-value” and the statistical results of categorical variables ^b^ were expressed as “OR (95%CI) p-value”.

Chi-square analysis and ANOVA analysis were performed to compare the pregnancy outcomes among the survivors with different treatment modalities (24: 39: 33). The statistical results were expressed as P ^c^.

OR, odds ratio; CI, confidence interval; PSM, propensity score matching.

### Impact of the Time Interval between Conception and Thyroidectomy

The survivors were trisected according to the time interval between conception and thyroidectomy: Group T1 (n =32, 14.15 [9.63-18.36] months), Group T2 (n =32, 33.50 [25.59-37.98] months), Group T3 (n =32, 66.83 [54.37-80.59] months). Compared with Group T1, no differences in pregnancy outcomes were found in Group T2 and T3. There were no statistically significant change trends in the trend test (**Table 6**).

**Table 6:** Impact of the time interval between conception and thyroidectomy.

	*P*			
	Group T1 (n=32)	Group T2 (n=32)	Group T3 (n=32)	*P for trend*
Conception time since thyroidectomy (month)^a^	14.15 (9.63-18.36)	33.50 (25.59-37.98)	66.83 (54.37-80.59)	
**Maternal outcomes**				
Postpartum hemorrhage (≧1000 mL)	1 (Referent)	0.996	>0.999	0.879
Preterm delivery	1 (Referent)	0.562	>0.999	0.922
Pre-eclampsia	1 (Referent)	0.996	0.644	0.430
Gestational diabetes mellitus	1 (Referent)	0.066	0.066	0.107
A1	1 (Referent)	0.180	0.110	0.139
A2	1 (Referent)	0.996	0.996	0.606
Late miscarriage	1 (Referent)	0.998	>0.999	0.831
Caesarean section	1 (Referent)	0.777	0.295	0.229
Anemia	1 (Referent)	0.400	0.400	0.464
Weight gain (kg)	0 (Referent)	0.425	0.884	0.993
Blood pressure in third trimester of pregnancy (mmHg)				
Systolic blood pressure	0 (Referent)	0.501	0.153	0.153
Diastolic blood pressure	0 (Referent)	0.454	0.404	0.441
**Neonatal outcomes**				
Macrosomia	1 (Referent)	0.998	>0.999	0.831
Small for gestational age	1 (Referent)	0.996	>0.999	0.879
Fetal death	1 (Referent)	0.996	>0.999	0.879
Malformation	1 (Referent)	0.998	0.998	0.998
Placental weight (g)	0 (Referent)	0.175	0.104	0.128
Birthweight (g)	0 (Referent)	0.046	0.599	0.833
Birth length (cm)	0 (Referent)	0.310	0.899	0.753

Data were presented as ^a^ median (first quartile, third quartile).

## Discussion

In this PS-matched cohort study, only reduced weight gain, decreased placental weight, and a lower risk of macrosomia were found in the survivors. Overall, no significant increased adverse pregnancy outcomes were detected in women with treated papillary thyroid cancer.

A higher incidence of pre-pregnancy hypertension and diabetes mellitus had previously been found in thyroid cancer survivors by two national database studies (13, 14). Nonetheless, the mean age of the survivors was much higher than that of the controls in those two studies. In our study, the survivors were also older than the controls before PSM (P=0.032). However, the baseline characteristics including the incidence of pre-pregnancy hypertension and pre-pregnancy diabetes mellitus were consistent between the two groups after PSM.

No significant difference was found in TSH, and the TSH of most survivors was not low. Therefore, the effects of TSH suppressive therapy on pregnancy outcomes could not be assessed in the present study. TPO-Ab levels and the incidence of abnormal TPO-Ab (≧60 U/mL) were higher in survivors (the mean values of TPO-Ab were still within the reference range). One possible explanation is that the release of thyroid antigens may occur during thyroidectomy and induce thyroid autoantibodies.^21^ Higher levels of fT4 were found in the survivors. Although there was a statistical significance (P<0.001), the difference was small (1 pmol/mL). In addition, we should interpret the results with caution, because the effect of diurnal variation (since patients’ blood samples were generally drawn between 0800 and 1130 hours) and CV (<4% for both intra- and inter-assay CV) could not be determined.

According to the results, there were no significant differences between women with a history of thyroid cancer and controls in adverse pregnancy outcomes, including pre-eclampsia, GDM, preterm delivery, and late miscarriage. These findings are consistent with two national database-based studies (13, 14) and three smaller-sized studies (15–17). The Korean study by Cho et al. (13) found a higher risk of PPH in the survivors, but no such difference was noted in our study. Different diagnostic standards, regions, and healthcare quality may have contributed to unmeasured confounding factors in the database-based studies, especially when based on ICD-10 diagnosis codes, which is mainly used for insurance and reimbursement purposes. In addition, postpartum blood loss is usually subjectively estimated and inaccurate. Consistent with our results, a recent study more accurately evaluated PPH with original records of blood loss amount and found no difference between thyroid cancer survivors and controls (16). It has been reported that RAIT is associated with the decrease of hemoglobin that is thought to be one of the risk factors for PPH. However, our result showed no difference in anemia risk between the two groups. Given that the interval between RAIT and conception is rather long in our study (34.70 [19.15–59.93] months), it might already have little effect on hemoglobin and PPH.

Interestingly, reduced weight gain, decreased placental weight, and a lower risk of macrosomia were found in the survivors. Initially, we thought that the changes in metabolic status caused by thyroid cancer treatment and TSH suppressive therapy altered the body’s metabolic status and reduced maternal, infant, and placental weight gain. However, TPO-Ab may also have played a role, as recent studies have shown that placental and fetal birth weights were lower in TPO-Ab-positive subjects (18–20). Therefore, we adjusted for fT4 and TPO-Ab separately. However, the differences were still significant after adjustment. This suggests the role of factors other than thyroid function. Other studies have confirmed that thyroid cancer survivors experienced greater psychological stress during pregnancy (21, 22). Moreover, a prospective cohort including 1,308 women found that stress and anxiety were associated with lower gestational weight gain (23). Therefore, the mental health status of thyroid cancer survivors may account for the results of our study but requires further research.

The survivors had reduced gestational weight gain, but it was still within the recommended range by the American Institute of Medicine (24). Although it has been reported that gestational weight gain may be related to the risk of pregnancy complications (25, 26), no increased incidence of major adverse pregnancy outcomes were detected among the survivors in our study.

The placental weight difference (9 g) was small, although statistically different. Moreover, since there was no statistically significant difference in birthweight or birthweight/placental weight (BW/PW, the ratio of fetal birth weight to placental weight, which indicates how successfully the placenta has adapted to the growing needs of the fetus [data not shown]), we believe that these carry little clinical significance.

Subgroup analysis showed no differences in pregnancy outcomes between survivors and the corresponding PS-matched controls. However, the significant differences in gestational weight gain, placental weight and the incidence of macrosomia between the survivors and the controls no longer exist, which may be caused by the reduced sample size. In terms of RAIT, the number of included women was small but is consistent with a large-scale real-world cohort study, thereby indicating that RAIT did not appear to be associated with increased adverse pregnancy outcomes (27).

Our study on time interval between conception and thyroidectomy found no significant differences or change trends among trisected time interval groups in pregnancy outcomes. However, due to the relatively large overall time interval and small sample size, this requires more large-scale studies to confirm.

### Strength and Limitation

Our study has some limitations. First, compared with studies based on national databases, the sample size was small, which limited the ability to detect minor differences. Second, women in our cohort were mainly from a tertiary referral hospital, limiting the generalizability of results. Third, all included survivors had papillary thyroid cancer and are therefore not representative of other pathological types. Last, thyroid function was not checked after 10–14 weeks of pregnancy.

The strengths of our study are as follows: First, confounders between the two groups were controlled as much as possible using PSM and logistic regression. Second, based on the original EMR, our data are more reliable than that from national databases based on ICD diagnosis codes alone. Third and perhaps the greatest strength of our study was that we provided detailed information on the pathological type, treatment, and gestational thyroid function of each thyroid cancer survivor. We believe that this study contributes to an improved understanding of the impact of treated thyroid cancer on pregnancy outcomes.

## Conclusion

In this retrospective cohort study involving 96 thyroid cancer survivors and 192 PS-matched control women, a history of papillary thyroid cancer was not associated with adverse pregnancy outcomes. These findings provide reassurance for women of childbearing age with a history of treated papillary thyroid cancer.

## Data Availability Statement

The raw data supporting the conclusions of this article will be made available by the authors, without undue reservation.

## Ethics Statement

The studies involving human participants were reviewed and approved by Ethics Committee of West China Second University Hospital of Sichuan University (No.2021098). Written informed consent for participation was not required for this study in accordance with the national legislation and the institutional requirements.

## Author Contributions

QC and WH made substantial contributions to the study’s conception and design, acquisition of data, and analysis. QC, HZ, and JZ made significant contributions to the interpretation of data and drafting of the manuscript. JZ made contributions to the acquisition of data. HZ and YL were involved in revising it critically for important intellectual content. All the authors have participated sufficiently in the work to take public responsibility for appropriate portions of the content and agree to be accountable for all aspects of the work to ensure that questions related to the accuracy or integrity of any part of the work are appropriately investigated and resolved.

## Conflict of Interest

The authors declare that the research was conducted in the absence of any commercial or financial relationships that could be construed as a potential conflict of interest.

## Publisher’s Note

All claims expressed in this article are solely those of the authors and do not necessarily represent those of their affiliated organizations, or those of the publisher, the editors and the reviewers. Any product that may be evaluated in this article, or claim that may be made by its manufacturer, is not guaranteed or endorsed by the publisher.

## Supplementary Material

Supplementary Figure S1
**(A–C)**. Directed acyclic graph illustrating confounder selection. IVF, *in vitro* fertilization; BMI, body mass index; fT4, free thyroxine; TSH, thyroid-stimulating hormone; TPO-Ab, thyroid peroxidase antibodies; GDM, gestational diabetes mellitus; PPH, postpartum hemorrhage.

## References

[1] Lim H Devesa SS Sosa JA Check D Kitahara CM. Trends in Thyroid Cancer Incidence and Mortality in the United States, 1974-2013. JAMA (2017) 317(13):1338–48. doi: 10.1001/jama.2017.2719 PMC8216772 28362912

[2] Kitahara CM Sosa JA. The Changing Incidence of Thyroid Cancer. Nat Rev Endocrinol (2016) 12(11):646–53. doi: 10.1038/nrendo.2016.110 PMC10311569 27418023

[3] Park S Oh C-M Cho H Lee JY Jung K-W Jun JK. Association Between Screening and the Thyroid Cancer “Epidemic” in South Korea: Evidence From a Nationwide Study. BMJ (Clinical Res Ed) (2016) 355:i5745. doi: 10.1136/bmj.i5745 PMC5130923 27903497

[4] Haddad RI Nasr C Bischoff L Busaidy NL Byrd D Callender G. NCCN Guidelines Insights: Thyroid Carcinoma, Version 2.2018. J Natl Compr Canc Netw (2018) 16(12):1429–40. doi: 10.6004/jnccn.2018.0089 30545990

[5] Haugen BR Alexander EK Bible KC Doherty GM Mandel SJ Nikiforov YE. 2015 American Thyroid Association Management Guidelines for Adult Patients With Thyroid Nodules and Differentiated Thyroid Cancer: The American Thyroid Association Guidelines Task Force on Thyroid Nodules and Differentiated Thyroid Cancer. Thyroid (2016) 26(1):1–133. doi: 10.1089/thy.2015.0020 26462967 PMC4739132

[6] Luster M Clarke SE Dietlein M Lassmann M Lind P Oyen WJG. Guidelines for Radioiodine Therapy of Differentiated Thyroid Cancer. Eur J Nucl Med Mol Imaging (2008) 35(10):1941–59. doi: 10.1007/s00259-008-0883-1 18670773

[7] Alexander EK Pearce EN Brent GA Brown RS Chen H Dosiou C. 2017 Guidelines of the American Thyroid Association for the Diagnosis and Management of Thyroid Disease During Pregnancy and the Postpartum. Thyroid (2017) 27(3):315–89. doi: 10.1089/thy.2016.0457 28056690

[8] Alexander EK Marqusee E Lawrence J Jarolim P Fischer GA Larsen PR. Timing and Magnitude of Increases in Levothyroxine Requirements During Pregnancy in Women With Hypothyroidism. N Engl J Med (2004) 351(3):241–9. doi: 10.1056/NEJMoa040079 15254282

[9] Mannisto T Mendola P Grewal J Xie Y Chen Z Laughon SK. Thyroid Diseases and Adverse Pregnancy Outcomes in a Contemporary US Cohort. J Clin Endocrinol Metab (2013) 98(7):2725–33. doi: 10.1210/jc.2012-4233 PMC3701274 23744409

[10] Medici M Korevaar TI Schalekamp-Timmermans S Gaillard R de Rijke YB Visser WE. Maternal Early-Pregnancy Thyroid Function is Associated With Subsequent Hypertensive Disorders of Pregnancy: The Generation R Study. J Clin Endocrinol Metab (2014) 99(12):E2591–8. doi: 10.1210/jc.2014-1505 25157540

[11] Korevaar TI Schalekamp-Timmermans S de Rijke YB Visser WE Visser W de Muinck Keizer-Schrama SM. Hypothyroxinemia and TPO-Antibody Positivity Are Risk Factors for Premature Delivery: The Generation R Study. J Clin Endocrinol Metab (2013) 98(11):4382–90. doi: 10.1210/jc.2013-2855 24037884

[12] Ceccarelli C Bencivelli W Morciano D Pinchera A Pacini F. 131I Therapy for Differentiated Thyroid Cancer Leads to an Earlier Onset of Menopause: Results of a Retrospective Study. J Clin Endocrinol Metab (2001) 86(8):3512–5. doi: 10.1210/jcem.86.8.7719 11502772

[13] Cho GJ Kim SY Lee HC Lee KM Han SW Oh MJ. Risk of Adverse Obstetric Outcomes and the Abnormal Growth of Offspring in Women With a History of Thyroid Cancer. Thyroid (2019) 29(6):879–85. doi: 10.1089/thy.2018.0283 PMC8917897 30957663

[14] Spiegel E Spence AR Czuzoj-Shulman N Abenhaim HA. Pregnancy Outcomes After Thyroid Cancer. J Perinat Med (2019) 47(7):710–6. doi: 10.1515/jpm-2019-0039 31323010

[15] Boucek J de Haan J Halaska MJ Plzak J Van Calsteren K de Groot CJM. Maternal and Obstetrical Outcome in 35 Cases of Well-Differentiated Thyroid Carcinoma During Pregnancy. Laryngoscope (2018) 128(6):1493–500. doi: 10.1002/lary.26936 28988434

[16] Liu D Wei Y Zhao Y Li R Yan J Qiao J. Obstetric Outcomes in Thyroid Cancer Survivors: A Retrospective Cohort Study. Int J Gynaecol Obstet (2020) 16:119–24. doi: 10.1002/ijgo.13571 33368229

[17] Clark H Kurinczuk JJ Lee AJ Bhattacharya S. Obstetric Outcomes in Cancer Survivors. Obstet Gynecol (2007) 110(4):849–54. doi: 10.1097/01.AOG.0000284458.53303.1c 17906019

[18] Ushijima J Furukawa S Sameshima H. The Presence of Thyroid Peroxidase Antibody Is Associated With Lower Placental Weight in Maternal Thyroid Dysfunction. Tohoku J Exp Med (2019) 249(3):231–6. doi: 10.1620/tjem.249.231 31776300

[19] Chen L-M Zhang Q Si G-X Chen Q-S Ye E-l Yu L-C. Associations Between Thyroid Autoantibody Status and Abnormal Pregnancy Outcomes in Euthyroid Women. Endocrine (2015) 48(3):924–8. doi: 10.1007/s12020-014-0420-x 25209893

[20] Plowden TC Schisterman EF Sjaarda LA Perkins NJ Silver R Radin R. Influence of Positive Serum Thyroid Peroxidase Antibody on Pregnancy Outcomes. Am J Obstet Gynecol (2017) 217(6):697.e1–e7. doi: 10.1016/j.ajog.2017.09.001 PMC5712235 28917612

[21] Schover LR Rybicki LA Martin BA Bringelsen KA. Having Children After Cancer. A Pilot Survey of Survivors' Attitudes and Experiences. Cancer (1999) 86(4):697–709. doi: 10.1002/(SICI)1097-0142(19990815)86:4<697::AID-CNCR20>3.0.CO;2-J 10440699

[22] Bresner L Banach R Rodin G Thabane L Ezzat S Sawka AM. Cancer-Related Worry in Canadian Thyroid Cancer Survivors. J Clin Endocrinol Metab (2015) 100(3):977–85. doi: 10.1210/jc.2014-3169 PMC4333046 25393643

[23] Harvey MW Braun B Ertel KA Pekow PS Markenson G Chasan-Taber L. Stress and Anxiety are Associated With Lower Gestational Weight Gain in Hispanic Women. Womens Health Issues (2020) 30(6):409–15. doi: 10.1016/j.whi.2020.08.003 PMC7704913 32994129

[24] Medicine IO. Weight Gain During Pregnancy: Reexamining the Guidelines. Washington (DC): National Academies Press (2009). 20669500

[25] Mamun AA Mannan M Doi SAR. Gestational Weight Gain in Relation to Offspring Obesity Over the Life Course: A Systematic Review and Bias-Adjusted Meta-Analysis. Obes Rev (2014) 15(4):338–47. doi: 10.1111/obr.12132 24321007

[26] Goldstein RF Abell SK Ranasinha S Misso M Boyle JA Black MH. Association of Gestational Weight Gain With Maternal and Infant Outcomes: A Systematic Review and Meta-Analysis. JAMA (2017) 317(21):2207–25. doi: 10.1001/jama.2017.3635 PMC5815056 28586887

[27] Kim HO Lee K Lee SM Seo GH. Association Between Pregnancy Outcomes and Radioactive Iodine Treatment After Thyroidectomy Among Women With Thyroid Cancer. JAMA Intern Med (2020) 180(1):54–61. doi: 10.1001/jamainternmed.2019.4644 31633736 PMC6806426

