# Supplementary figures and images for: Pregnancy Outcomes in Thyroid Cancer Survivors: A Propensity Score-Matched Cohort Study
Source: Front Endocrinol (Lausanne). 2022 Feb 17;13:816132. doi: 10.3389/fendo.2022.816132 (PMC8893319; doi:10.3389/fendo.2022.816132)

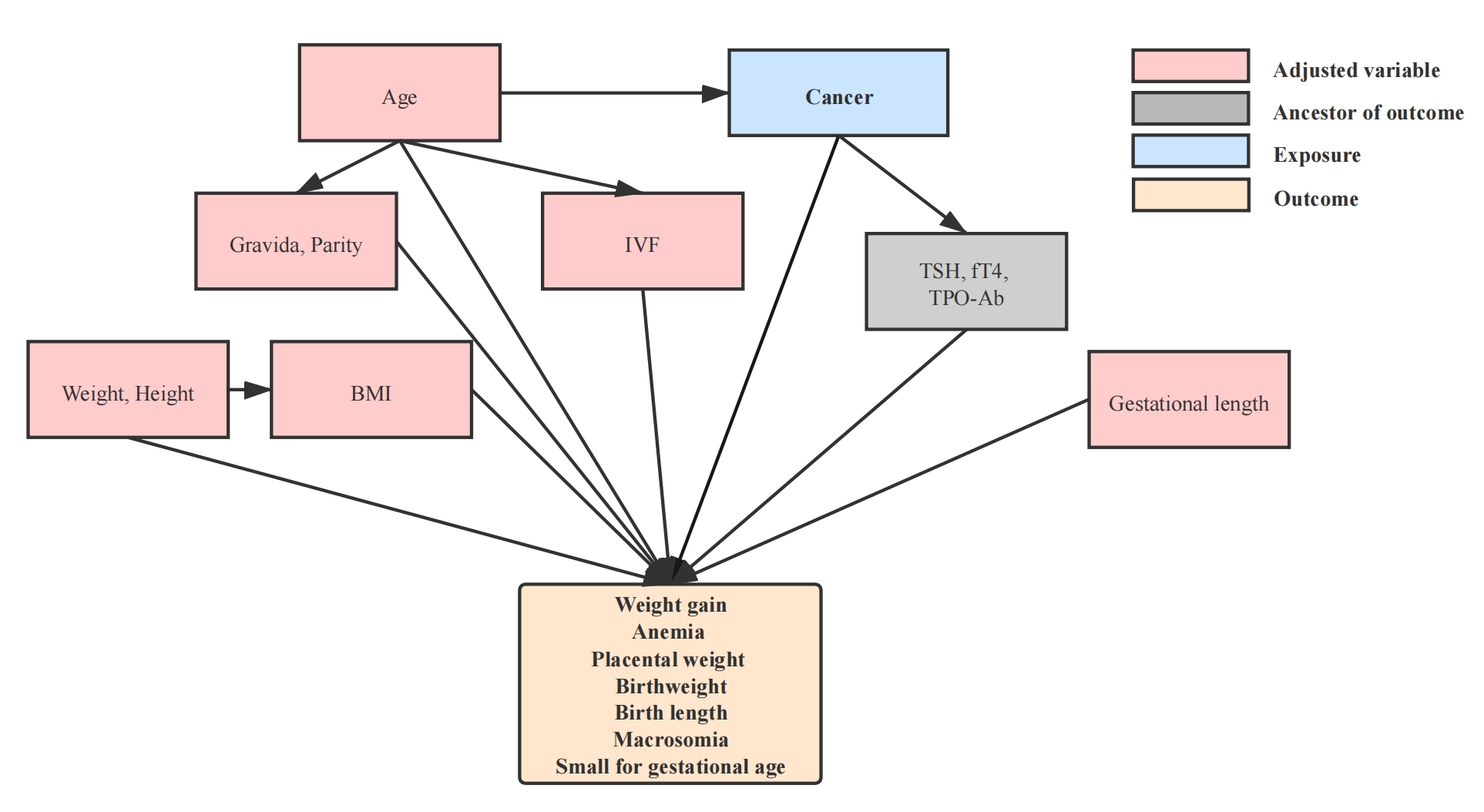

Supplement: Supplementary Figure S1 — (A–C). Directed acyclic graph illustrating confounder selection. IVF, in vitro fertilization; BMI, body mass index; fT4, free thyroxine; TSH, thyroid-stimulating hormone; TPO-Ab, thyroid peroxidase antibodies; GDM, gestational diabetes mellitus; PPH, postpartum hemorrhage. [file Image_1.tif]

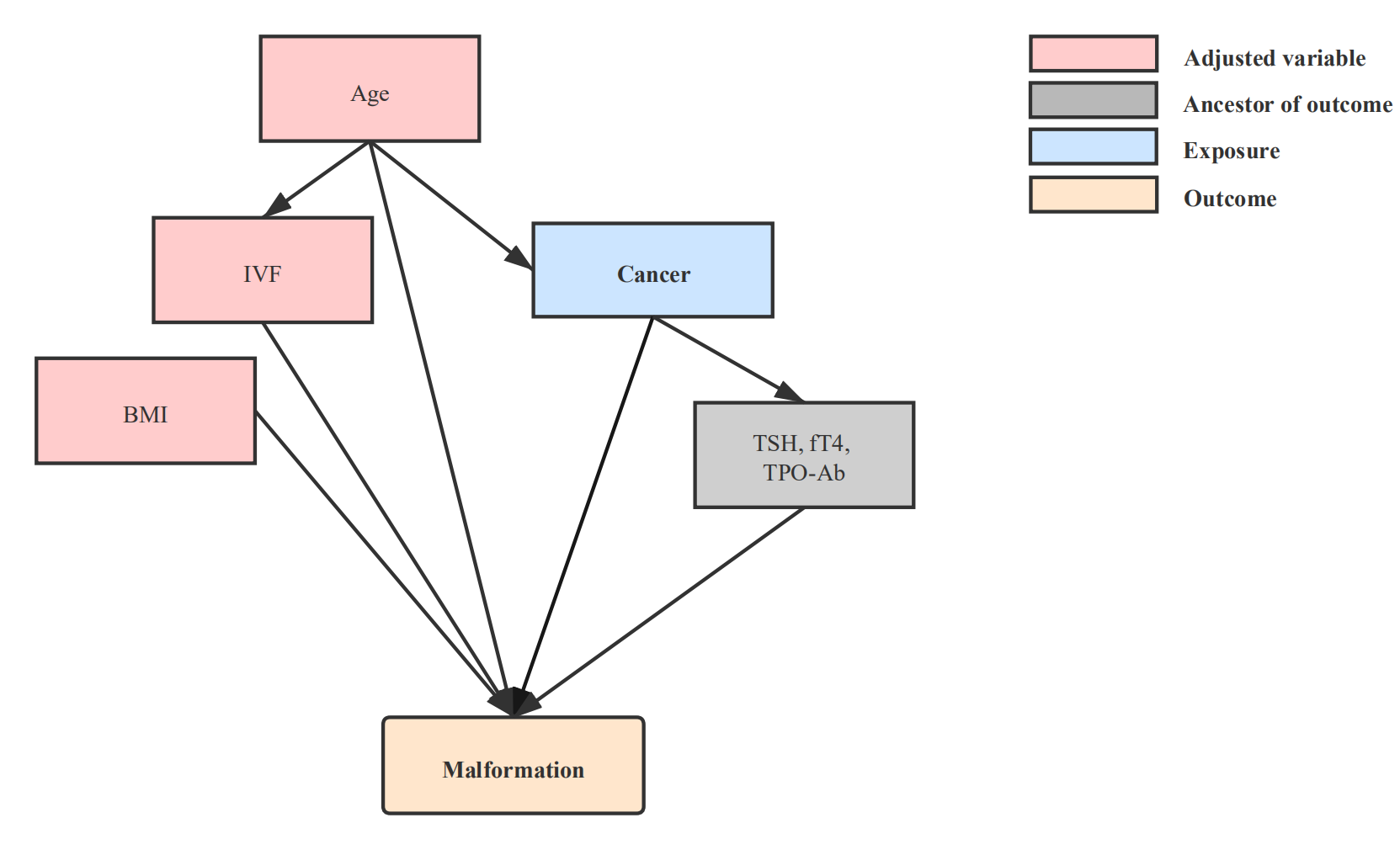

Supplement: Supplementary file 2 [file Image_2.tif]

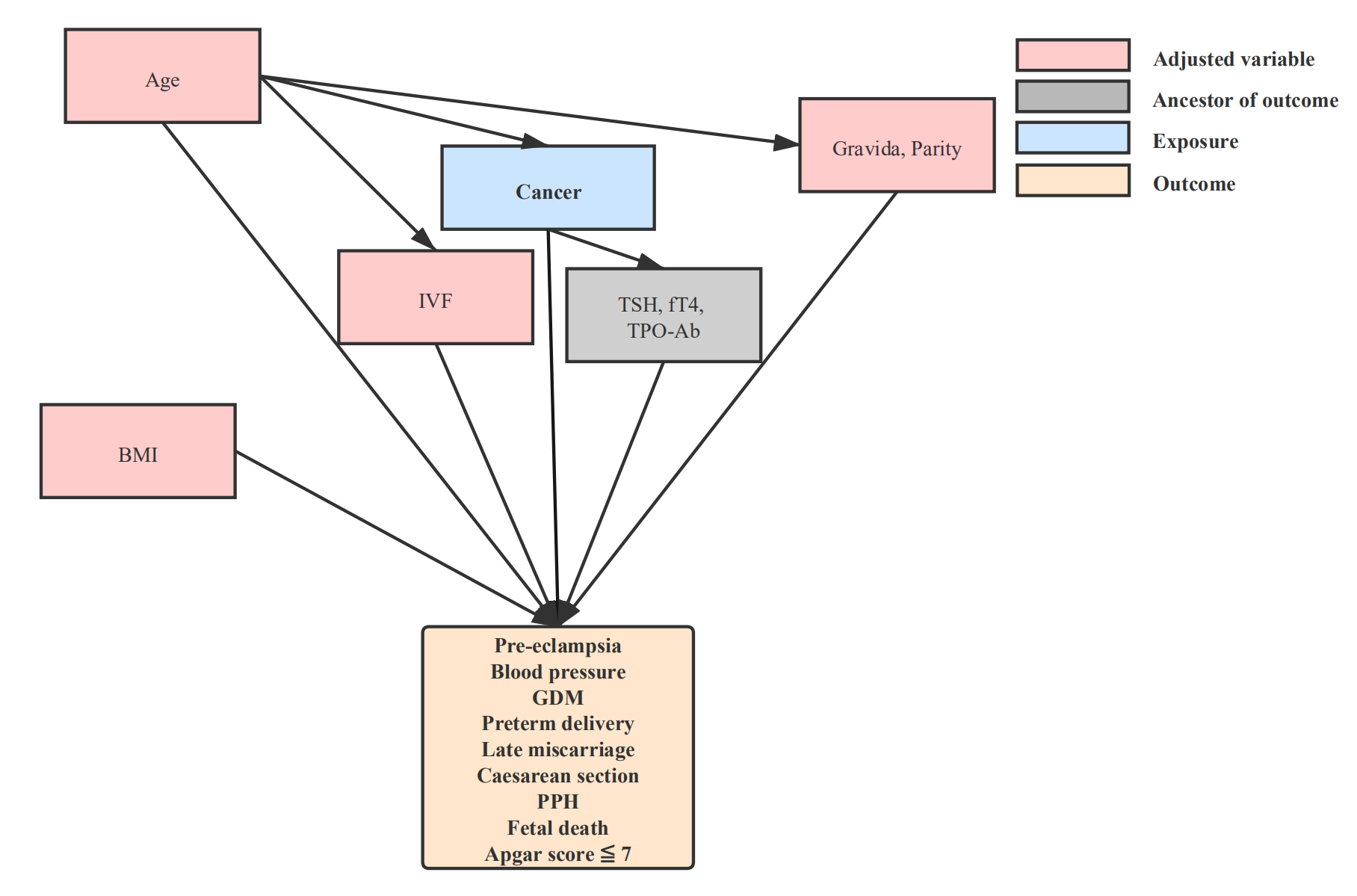

Supplement: Supplementary file 3 [file Image_3.tif]
