# Supplementary material for: Pregnancy Outcomes in Thyroid Cancer Survivors: A Propensity Score-Matched Cohort Study
Source: Front Endocrinol (Lausanne). 2022 Feb 17;13:816132. doi: 10.3389/fendo.2022.816132 (PMC8893319; doi:10.3389/fendo.2022.816132)
Supplement: Supplementary file 4 [file Table_1.docx]

Table S1. Information about thyroid cancer therapy of the survivors.

|  |  | Number | Conception time since thyroidectomy (month) a | Conception time since last RAIT (month) a | LT4 dosage  (μg per day) ^a^ | RATI dosage  (millicurie) ^b^ |
| --- | --- | --- | --- | --- | --- | --- |
| Pathological type | |  |  |  |  |  |
|  | Papillary thyroid cancer | 96 | 33.50 (18.36-54.37) | 34.70 (19.15-59.93) | 122.5 (100-125) | 108±28 |
|  | Other types (MTC, ATC or other DTC) | 0 | - | - | - | - |
| Treatment modality | |  |  |  |  |  |
|  | Hemi‐thyroidectomy (without RAIT) | 24 | 23.95 (14.63-44.27) | - | 87.5 (75-110) | - |
|  | Total-thyroidectomy (without RAIT) | 39 | 28.67 (12.93-54.40) | - | 125 (100-125) | - |
|  | Total-thyroidectomy combined RAIT | 33 | 44.37 (21.72-67.45) | 34.70 (19.15-59.93) | 125 (100-143.75) | 108±28 |

Data are presented as ^a^ median (first quartile, third quartile), ^b^ mean±standard deviation), ^c^ number (the percentage).

MTC, medullary thyroid carcinoma; ATC, anaplastic thyroid cancer; DTC, differentiated thyroid carcinoma; RAIT, radioactive iodine therapy.
